# Effects of supportive and minimalist footwear on standing balance and walking stability in older women

**DOI:** 10.1186/s13047-023-00634-y

**Published:** 2023-06-19

**Authors:** Ameer Nor Azhar, Shannon E. Munteanu, Hylton B. Menz

**Affiliations:** Discipline of Podiatry, School of Allied Health, Human Services and Sport, La Trobe University, Melbourne, VIC 3086 Australia

**Keywords:** Ageing, Falls, Postural balance, Footwear

## Abstract

**Background:**

Footwear has been shown to influence balance and is an important consideration in relation to the prevention of falls. However, it remains unclear as to what type of footwear is most beneficial for balance in older people: sturdy, supportive footwear, or minimalist footwear to maximise plantar sensory input. The objectives of this study were therefore to compare standing balance and walking stability in older women wearing these two footwear styles, and to investigate participants’ perceptions in relation to comfort, ease of use and fit.

**Methods:**

Older women (*n* = 20) aged 66 to 82 years (mean 73.4, SD 3.9) performed a series of laboratory tests of standing balance (eyes open and closed on floor and foam rubber mat, near tandem standing) and walking stability (treadmill, level and irregular surface) using a wearable sensor motion analysis system. Participants were tested wearing supportive footwear (incorporating design features to improve balance) and minimalist footwear. Perceptions of the footwear were documented using structured questionnaires.

**Results:**

There were no statistically significant differences in balance performance between the supportive and minimalist footwear. Participants perceived the supportive footwear to be significantly more attractive to self and others, easier to put on and off but heavier compared to the minimalist footwear. Overall comfort was similar between the footwear conditions, although the supportive footwear was reported to be significantly more comfortable in the heel, arch height, heel cup, heel width and forefoot width regions. Eighteen participants (90%) reported that they felt more stable in the supportive footwear and 17 (85%) reported that they would consider wearing them to reduce their risk of falling.

**Conclusion:**

Balance performance and walking stability were similar in supportive footwear designed to reduce the risk of falling and minimalist footwear, although participants preferred the supportive footwear in relation to aesthetics, ease of use, comfort and perceived stability. Prospective studies are now required to ascertain the longer-term advantages and disadvantages of these footwear styles on comfort and stability in older people.

**Trial registration:**

Australian New Zealand Clinical Trials Registry. ACTRN12622001257752p, 20/9/2022 (prospectively registered).

## Background

Falls in older people are extremely common [1]. Footwear has the potential to influence balance in either a harmful or favourable manner, and is therefore an important consideration in relation to the prevention of falls. Laboratory-based studies have shown that elevated heels [2–5], cushioned soles [3, 4, 6] and inadequate fixation [7] are detrimental to balance. This is of particular concern for older women, as many styles of female footwear incorporate these potentially hazardous features. For example, women are more likely to wear shoes with high heels [5] or slippers [7] than men.

Footwear with high collars [3, 8–12], firm soles [4, 9, 10, 13] and adequate fixation [13–15] is considered to be beneficial. It has therefore been suggested that older people at risk of falling should wear supportive shoes with a low, broad heel, a thin, firm midsole, a high collar and a textured, slip-resistant outersole [16]. However, it has also been suggested that because somatosensory feedback from the plantar surface of the foot plays an important role in balance, older people should wear shoes that mimic barefoot walking as closely as possible [17]. Indeed, using various types of balance testing apparatus, balance has been found to be better barefoot than wearing shoes [18, 19], with minimalist shoes being better for balance than barefoot [20] or conventional, supportive footwear [21].

In a previous study, we found that prototype footwear improved balance compared to flexible footwear when older women performed a tandem walk test, as evidenced by a narrower step width and decreased sway at completion of the task [12]. However, the prototype footwear was deemed to be less attractive, more uncomfortable, less well-fitted and harder to put on and off compared to their own footwear, and half rated the appearance of the prototype footwear as problematic. Clearly, aesthetics plays an important role in the selection of footwear by older women [22]. Therefore, the objectives of this study were to compare balance ability and walking stability in older women while wearing more aesthetically appealing supportive and minimalist footwear, and to investigate older womens’ perceptions of the two types of footwear.

## Methods

### Participants

Older women (aged 65 or over) were recruited via completing a mail-out using a database of people who had been attending the La Trobe University Podiatry Clinic for treatment of foot problems. From the mail-out, candidates were screened through a telephone call, after the screening process, 20 participants were recruited. Eligible participants needed to be female, over 65 years of age, able to walk household distances (more than 50 m) without a walking aid, capable of understanding the English language in verbal and written form, and not have a neurodegenerative condition (e.g., Parkinson’s disease), lower limb amputation, or have undergone foot and ankle surgery in the previous 3 months. Ethical approval was granted from the La Trobe University Human Ethics Committee (HEC22227), and written informed consent was obtained from all participants. This study was conducted as part of a larger series of studies [12, 13].

An a priori sample size calculation (using G*Power version 3.1.9.4, Kiel, Germany) estimated that 19 participants were required to provide 80% power to detect a large effect size (*d* = 0.70) between the two footwear conditions, with statistical significance for hypothesis tests set at *p* < 0.05 (two-tailed). The large effect size was justified on the basis of our previous footwear and balance study identifying large effect sizes for the difference between step width and end sway when wearing the first prototype balance-enhancing shoes compared to flat, flexible shoes [12].

### Questionnaire and clinical assessment

A self-completion questionnaire was administered which included basic participant, demographic and medical history data (age, a checklist of common medical conditions and medication usage), falls in the previous 12 months, fear of falling (using the Falls Efficacy Scale International [23]), general health (using the Short Form-12 Version 2 survey [24]) and physical activity (using the Incidental and Planned Exercise Questionnaire [25]). The presence and severity of foot pain was documented using the Manchester Oxford Foot Questionnaire [26], using the total index, pain, walking / standing and social interaction scores.

### Falls risk assessment

Risk of falling was evaluated using the validated QuickScreen^©^ tool [27], which consists of eight measures: (i) previous falls, (ii) total medications, (iii) use of psychoactive medications, (iv) visual acuity (using a 10% low contrast letter chart), (v) touch sensation (using a Semmes–Weinstein-type pressure aesthesiometer applied to the lateral malleolus), (vi) the sit to stand test (using a 430 mm high chair without armrests, five times as fast as possible with arms folded), (vii) the near tandem stand test (eyes closed, with feet separated laterally by 25 mm and the heel of the front foot 25 mm anterior to the great toe of the back foot) and (viii) the alternate step test (alternatively placing the whole left and right feet as fast as possible onto a 190 mm high and 400 mm deep step eight times). Each of these measures was dichotomised using established cut-points [27].

### Balance and walking stability assessment

**Fig. 1:**
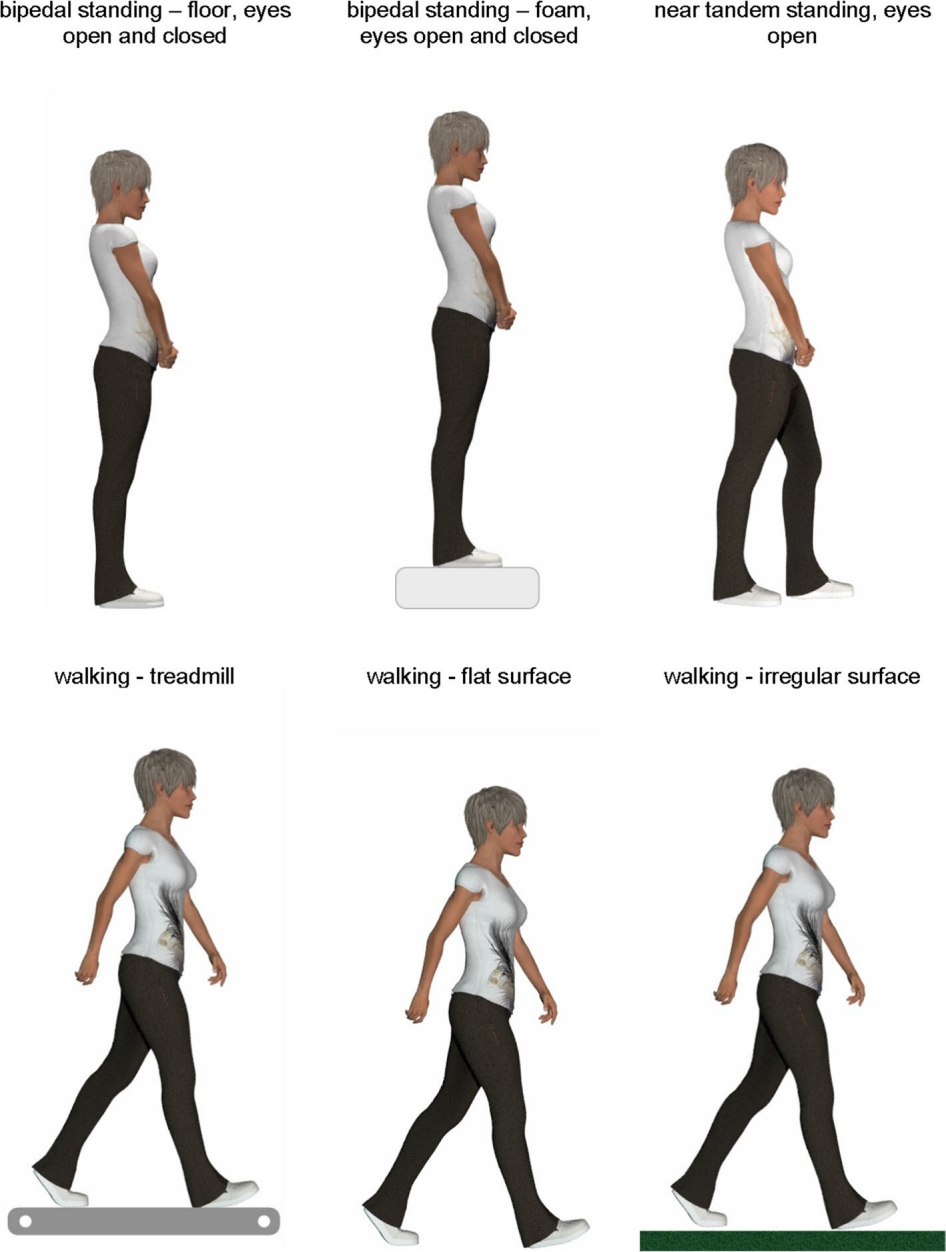
Balance and walking stability tests. See text for explanation

We measured area (in centimetres) of postural sway and walking stability using a wearable sensor (dimensions: 50 × 70 × 20 mm; mass: 35 g; Gyko, Microgate, Bolzano, Italy) which was attached to participants at the level of the thoracic spine using a special harness and documented movements up to 16 g and angular velocities of up to 2000°/sec with an acquisition frequency of 1000 Hz. The reliability of the Gyko system has been previously reported [28, 29]. We measured bipedal standing (floor and foam [460 × 460 × 130 mm], eyes open and closed), near-tandem standing (feet separated laterally by 25 mm and the heel of the front foot 25 mm anterior to the great toe of the back foot with eyes open), and walking on a treadmill, flat surface and irregular surface (foam plates randomly placed covered with artificial grass) (see Fig. 1). For the postural sway tests, we recorded for 30 s. For the treadmill walking, speed was set at 4 km/h, which is the average speed of a 60 + year-old woman [30]. However, we found that three women (15%) were unable to comfortably walk at this speed, so we tested them at 2.2 km/h and 1.2 km/h, respectively (two out of the three participants were tested at 1.2 km/h). Treadmill walking was recorded for 60 s, and we allowed participants to walk at their own speed for the flat and irregular surface, of which four trials were recorded of each over an eight metre distance.

### Footwear conditions

**Fig. 2:**
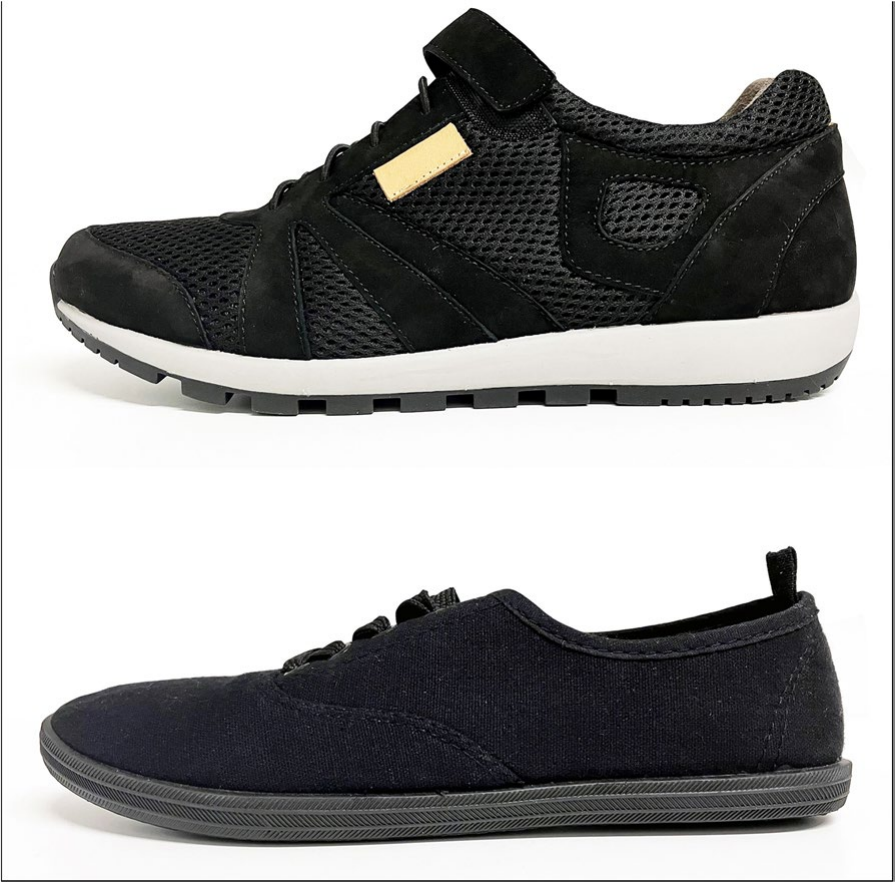
Supportive (top) and minimalist (bottom) footwear used in the study

Participants performed each of the balance and gait assessments when wearing supportive and minimalist footwear. The Brannock device^®^ was used to determine the appropriate size for the participants [31]. Order of testing was randomised (using Microsoft Excel, Microsoft Corp, Washington, USA) to avoid order (i.e., habituation or fatigue) effects. Figure 2 shows key features of the supportive and minimalist footwear.

The supportive footwear was based on an existing model (Ziera, Munro Footwear Group, Abbotsford, Australia) and was manufactured by Able Health (Sydney, Australia). The footwear had a firm (Shore A hardness 55 [32]) rubber sole of 20 mm thickness under the heel and 10 mm under the forefoot, laces plus Velcro^*®*^ fastening, and a firm heel counter. The weight of the supportive footwear was 313 to 342 gm across the size range. The outersole had a 10 degree bevel into the heel region [33, 34], grooves perpendicular to the sole (1.2 mm deep and 2.4 mm wide) across the heel surface area [35], and perpendicular grooves (5 mm deep and 12 mm wide) across the rest of the sole [36, 37]. A textured insole was also constructed from 4 mm thick ethyl vinyl acetate (Shore A 25 [32]) with dome-shaped projections (3 mm high and 8 mm diameter, Shore A 85 [32]) placed across the forefoot in a 15 mm diamond pattern and along the lateral border, extending to the heel. The textured insole was informed by previous studies reporting improvements on balance in older people when similar insoles were worn [12, 38, 39]. The shoes were similar to our previous study [12] but lacked the high ankle collar and were manufacturered with the aim to be more aesthetically pleasing than the first prototype.

The minimalist footwear (Kmart, Wesfarmers, Perth, Australia) had a canvas upper and rubber sole of uniform 10 mm heel and 5 mm forefoot outersole thickness and lace fixation, and a hardness of Shore A 35 [32]. The weight of the minimalist footwear was 191 to 258 gm across the size range. The minimalist footwear was chosen as a control condition as it had no features deemed to be either beneficial or detrimental to balance. The footwear met the criteria to be considered ‘minimalist’ outlined by the Esculier et al. [40] study, namely that it provided “minimal interference with the natural movement of the foot due to its high flexibility, low heel to toe drop, weight and stack height, and the absence of motion control and stability devices”.

### Footwear assessment

After balance and walking assessment, both types of footwear were assessed using questions selected from the Monitor Orthopaedic Shoes questionnaire [41] scored on a 100 mm visual analog scale. The selected questions were: (i) please mark on the following line how attractive you think the shoes are (with the anchors “extremely unattractive” and “extremely attractive”), (ii) please mark on the following line how attractive you think other people would think the shoes are (with the anchors “extremely unattractive” and “extremely attractive”), (iii) please mark on the following line how comfortable you think the shoes are (with the anchors “extremely uncomfortable” and “extremely comfortable)”, (iv) please mark on the following line how well you think the shoes fit you (using the anchors “poorest fit possible” and “best fit possible”), (v) please indicate how easy it is for you to don the shoes on and off (using the anchors “most difficult as possible” and “as easy as imaginable”) and (vi) please indicate how heavy the shoes are (using the anchors “extremely light” and “extremely heavy”). Participants were also asked whether they felt more balanced in the supportive footwear (on a 100 mm visual analog scale), would consider wearing them if they found to be beneficial for balance (with the options yes, no, or maybe), and whether the design could be improved (open-ended response).

Footwear comfort for both types of footwear was also assessed using the comfort scale described by Műndermann et al. [42] which enables the documentation of footwear comfort both overall and specific to heel cushioning, forefoot cushioning, medio-lateral control, arch height, heel cup fit, shoe heel width, shoe forefoot width, and shoe length. Participants were asked to rate the footwear on a 100 mm visual analog scale using the anchors “not comfortable at all” and “most comfortable condition imaginable”.

### Statistical analysis

Statistical analysis was undertaken using SPSS Version 29.0 (IBM, Armonk, NY, USA). Differences between the two footwear conditions (supportive footwear and minimalist footwear) were evaluated using paired samples *t*-tests. Level of significance was set at 0.05. Effect sizes for between-group comparisons were calculated using Cohen’s *d*, and were interpreted as follows: ≤ 0.01 = very small, > 0.01 to 0.20 = small, > 0.20 to 0.50 = medium, > 0.50 to 0.80 = large, > 0.80 to 1.20 = very large, and > 1.20 = huge [43].

## Results

### Participant characteristics

Participant characteristics are shown in Table 1. One participant had missing data due to a technical error for the postural sway on the floor with eyes closed (wearing minimalist shoes) test, five had missing data for the postural sway eyes closed on the foam test (five wearing minimalist shoes, due to an inability to complete the test), and one had missing data for the near tandem test (wearing minimalist shoes, due to an inability to complete the test). Those who were unable to complete the test were given the worst score of the remaining sample, as we have done previously [12].

**Table 1:** Participant characteristics. Values are n (%) unless otherwise stated

Age, mean (SD) years	73.4 (3.9)
Height, mean (SD) cm	159.6 (6.0)
Weight, mean (SD) kg	67.9 (14.7)
Body mass index, mean (SD) kg/m^2^	26.6 (5.1)
Major medical conditions	
Heart disease	2 (10)
Diabetes	1 (5)
Stroke	4 (20)
Osteoarthritis	10 (50)
High blood pressure	11 (55)
Short Form-12 Version 2^a^	
Role – physical, mean (SD)	46.8 (10.1)
Role – mental, mean (SD)	54.3 (9.2)
Incidental and Planned Exercise Questionnaire total, mean (SD) hours/week	34.4 (11.1)
QuickScreen falls risk assessment	
At least one falls risk factor	20 (100)
Fallen in past 12 months	4 (20)
Use of 4 or more medications	9 (45)
Use of psychotropic medications	1 (5)
Impaired visual acuity	20 (100)
Impaired peripheral sensation	3 (15)
Failed near tandem stance test	3 (15)
Failed alternate step test	6 (30)
Failed sit-to-stand test	2 (10)
Falls Efficacy Scale International, mean (SD)^b^	21.5 (5.2)
Manchester Oxford Foot Questionnaire^c^	
Total index score, mean (SD)	18.8 (21.3)
Pain, mean (SD)	22.8 (24.7)
Walking / standing, mean (SD)	18.4 (21.7)
Social interaction, mean (SD	14.4 (18.5)

^a^Score range from 0 to100; higher score indicates better function

^b^Score ranges from 16 to 64; higher score indicates greater fear (low 16–19, moderate 20–27, high 28–64)

^c^Score ranges from 0 to 100; higher score indicates greater pain

### Effects of footwear on balance

Results of the paired sample *t*-tests for the balance tests are shown in Table 2. Overall, there was no significant difference in postural sway eyes open on the floor (*d* = 0.14, small effect, *p* = 0.320), postural sway eyes open on the foam (*d* = 0.26, medium effect, *p* = 0.099), postural sway eyes closed on the foam (*d* = 0.13, small effect, *p* = 0.324) and near tandem stance (*d* = 0.19; small effect, *p* = 0.204). However, there was a tendency for better performances in the minimalist footwear than the supportive footwear for the postural sway eyes open on the floor, postural sway eyes closed on the floor, and near tandem tests (small to medium effect sizes).

**Table 2:** Differences in balance and gait patterns between the supportive footwear and minimalist footwear. Values are in centimetres

	Supportive footwear		Minimalist footwear		Cohen’s* d*	Interpretation	*P*-value
	mean (SD)	median (IQR)	mean (SD)	median (IQR)			
Balance							
Postural sway, eyes open on the floor	172 (335)	81 (77)	135 (152)	78 (88)	0.14	small	0.320*
Postural sway, eyes closed on the floor	88 (46)	74 (76)	68 (56)	47 (52)	0.36	medium	0.057*
Postural sway, eyes open on the foam	663 (619)	460 (566)	533 (385)	456 (434)	0.26	medium	0.099*
Postural sway, eyes closed on the foam	3,824 (8,146)	913 (737)	4,750 (6,062)	1,405 (13,202)	0.13	small	0.324†
Near tandem stance	929 (972)	429 (955)	760 (878)	514 (522)	0.19	small	0.204*
Walking stability							
Walking stability, treadmill	3,891 (2,917)	2,745 (2,175)	16,377 (53,923)	2,809 (2,134)	0.34	medium	0.157†
Walking stability, level surface	20,264 (16,077)	13,395 (12,703)	24,060 (28,598)	14,301 (10,661)	0.17	small	0.236†
Walking stability, irregular surface	21,812 (12,398)	17,364 (12,256)	29,463 (32,038)	16,526 (10,870)	0.32	medium	0.141†

*SD* Standard deviation, *IQR* Interquartile range

^*^Non-significant improvement with minimalist footwear

^†^Non-significant improvement with supportive footwear

### Effects of footwear on walking stability

Results of the paired sample *t*-tests for the balance tests are shown in Table 2. There was no significant difference in treadmill walking stability (*d* = 0.34, medium effect, *p* = 0.157), walking stability on the floor (*d* = 0.17, small effect, *p* = 0.236), or walking stability on the irregular surface (*d* = 0.32, medium effect, *p* = 0.141). However, there was a tendency for better performances in the supportive footwear than the minimalist footwear for the treadmill, floor and irregular surface walking tests (small to medium effect sizes).

### Perceptions of footwear

Participants’ perceptions of the supportive and minimalist footwear are shown in Table 3. Participants perceived the supportive footwear to be significantly more attractive to self (*d* = 0.72, large effect, *p* = 0.011), and others (*d* = 0.82, very large effect, *p* = 0.010), easier to put on and off (*d* = 0.77, large effect, *p* = 0.009), but marginally less comfortable (*d* = 0.14, small effect, *p* = 0.656) and heavier (*d* = 1.45, huge effect, *p* = 0.001), compared to the minimalist footwear. Overall comfort (*d* = 0.10, small effect, *p* = 0.752), forefoot cushioning comfort (*d* = 0.00, very small effect, *p* = 0.932) and shoe length comfort (*d* = 0.35, medium effect size, *p* = 0.182) was similar between the footwear conditions, although the supportive footwear was reported to be significantly more comfortable in relation to heel cushioning (*d* = 1.23, huge effect, *p <* 0.001), arch height (*d* = 1.45, huge effect, *p* < 0.001), heel cup fit (*d* = 0.96, very large effect, *p* = 0.004), shoe heel width (*d* = 0.71, large effect, *p* = 0.013) and shoe forefoot width (*d* = 0.63, large effect, *p* = 0.017) regions. Eighteen participants (90%) reported that they felt more stable in the supportive footwear and 17 (85%) reported that they would consider wearing them to reduce their risk of falling. When asked how best to improve the supportive footwear, removal of the insole projections was the most common recommendation (*n* = 9, 45%), although discomfort was thought to reduce over time (*n* = 3, 15%), and discomfort could be ameliorated by making the insole softer (*n* = 3, 15%) or by reducing the number of projections under the toes (*n* = 2, 10%). A wider selection of colours was also deemed important (*n* = 5, 25%), as was removal of the Velcro^*®*^ strap (*n* = 1, 5%) and the provision of a larger range of widths (*n* = 1, 5%).

**Table 3:** Differences in perceptions of supportive footwear and minimalist footwear. Values are mean (SD) mm from 100 mm visual analog scales. Higher scores represent greater perceived attractiveness, comfort, fit, ease of donning and doffing, heaviness and location of comfort

	Supportive footwear	Minimalist footwear	Cohen’s *d*	Interpretation	*P*-value
Monitor Orthopaedic Shoes Questionnaire^a^					
Attractiveness to self	80.0 (19.0)	62.1 (30.9)	0.72	large	0.011*
Attractiveness to others	77.2 (19.1)	56.9 (30.6)	0.82	very large	0.010*
Comfort	61.2 (25.4)	65.1 (30.2)	0.14	small	0.656
Fit	84.9 (10.3)	74.5 (24.8)	0.56	large	0.081
Ease of donning and doffing	90.3 (8.3)	80.4 (16.6)	0.77	large	0.009*
Heaviness	27.7 (18.5)	6.8 (9.6)	1.45	huge	0.001†
Comfort scale^b^					
Overall	64.6 (23.7)	62.0 (30.4)	0.10	small	0.752
Heel cushioning	83.5 (9.7)	53.8 (33.6)	1.23	huge	< 0.001*
Forefoot cushioning	52.2 (30.1)	52.1 (29.2)	0.00	very small	0.932
Medio-lateral control	76.0 (23.2)	56.9 (28.5)	0.75	large	0.048*
Arch height	79.2 (32.4)	43.0 (16.3)	1.45	huge	< 0.001*
Heel cup fit	87.0 (7.8)	64.8 (32.8)	0.96	very large	0.004*
Shoe heel width	85.9 (9.8)	73.4 (23.4)	0.71	large	0.013*
Shoe forefoot width	82.3 (17.2)	68.4 (26.9)	0.63	large	0.017*
Shoe length	83.8 (12.4)	78.9 (15.8)	0.35	medium	0.182

^*^Significant improvement with supportive footwear

^†^Significant improvement with minimalist footwear

^a^Score range from 0 to100; higher score indicates better function

^a^Score range from 0 to100; higher score indicates greater comfort

## Discussion

The primary objective of this study was to evaluate standing balance and walking stability in older women while wearing two different types of footwear: minimalist control footwear and supportive prototype footwear designed to reduce the risk of falling. Our findings indicate that standing balance or walking stability performance between the minimalist and supportive conditions were similar. However, there were trends which saw better standing balance performances in minimalist footwear compared to the supportive footwear, and there was a tendency for better walking stability performance in the supportive footwear compared to the minimalist footwear.

There are three main explanations for the lack of significant differences in postural sway and walking stability between the footwear conditions. First, the minimalist footwear we used as the control condition had no features considered to be beneficial to balance, but also had no features that were potentially hazardous. This is similar to our previous comparison of a prototype balance-enhancing shoe and flexible shoe [12]. Second, our supportive footwear lacked the high heel collar of our initial prototype, which was an attempt to make the shoe more aesthetically pleasing but may have impacted on its balance-enhancing function [12]. Third, participants were healthy and active considering their ages and were able to complete most, if not all tests with relative ease. More challenging tests could be employed for future studies which may result in greater differentiation between the two footwear conditions, although we acknowledge that the tests in the current study have previously been used to discriminate between different types of footwear [2, 3, 8].

The secondary objective of this study was to investigate older womens’ perceptions of the footwear. Participants perceived the supportive footwear to be significantly more attractive to self and others, and also to put on and off compared to the minimalist footwear. The supportive footwear was also perceived to be slightly less comfortable overall and considerably heavier than the minimalist footwear. Forefoot cushioning comfort and shoe length comfort was similar between the two footwear conditions, however the supportive footwear was reported to be significantly more comfortable in relation to heel cushioning, arch height, heel cup fit, heel width and shoe forefoot width regions. Ninety percent of participants felt more stable in the supportive prototype shoe and 17 (85%) reported that they would consider wearing them again to reduce their risk of falling.

When asked how best to improve the supportive footwear, removal of the insole projections was the most common recommendation (*n* = 9, 45%), although discomfort was thought to reduce over time by three of these participants. Two other changes to the insole projections were also recommended: making the insole softer, or reducing the number of projections under the toes was thought to reduce discomfort by three (15%) participants. Greater selection of colours was also deemed to be important by five (25%) participants as was the removal of the Velcro^®^ strap (one participant; 5%) and the provision of different width offerings (one participant; 5%). These findings are encouraging as changes to the insole and the projections can be easily made and materials used for the supportive shoe can be manufactured in several colours.

Our results are similar to our previous comparison of a prototype balance-enhancing shoe and flexible shoe [12], although the supportive footwear in the current study was deemed to be more attractive (to self and others) and easier to don and doff, probably because of the lower cut heel collar profile. In contrast to a previous study [21], we found no statistically significant differences in balance and walking stability between supportive and minimalist shoes, however this observation needs to considered in the context of key differences between the studies. We used wearable sensors to measure upper trunk movements while standing and walking, while Cudejko et al. [21] used a force plate to measure postural sway and a pressure plate to measure ‘dynamic’ stability, inferred by mean velocity and the maximum range of centre of pressure displacement in the mediolateral direction. Furthermore, their ‘conventional’ shoe had a much higher heel (1.25 inches, which equals 3.175 cm) than our supportive shoe (1 cm), and the authors suggested that the poorer performance of their conventional shoes may have been due to the higher heel shifting the total body center of mass anteriorly [21].

The findings of this study need to be interpreted in the context of several limitations and highlight that these data represent only a preliminary evaluation of the footwear given the relatively small sample. First, although previous research has shown that 5 weeks of habituation to new shoes does not significantly affect standing balance or gait patterns in older women [44], previous studies have used a habituation period of between 1 min [19, 20] and a few days [45]. In our study, participants were only provided with a brief period of time to acclimatise to the different footwear conditions before undertaking the balance tests. More time spent in the supportive footwear over a prolonged period would have allowed participants to acclimitise to the supportive devices in the shoe such as the textured insole. Furthermore, materials such as leather which are initially stiff when new would start to soften as the wearing process continues, allowing for a better wearing experience for the participant. Second, our supportive footwear is designed to be worn outdoors, and it has been shown that older people who fall indoors are more likely to be older, less physically active and have poorer general health [46]. It is therefore likely that indoor fallers would be better served by a supportive slipper rather than a conventional shoe [13]. Third, participants were not blinded to their intervention, so their maybe some bias in their responses to their perceptions of balance, which was reported to be better in the supportive shoes. Fourth, it would be of interest assess balance performance using the participants’ own footwear as a control, as this may improve the external validity of the study findings. Fifth, a limitation of the software we used required that the walking speed be prespecified for the treadmill walking tests, as the unit of analysis was time (60 s). We chose to set this at 4 km/h, which is the average speed of a 60 + year-old woman [14] but found that three women could not walk at this speed. In future studies, we recommend setting this to the participant’s comfortable speed. Sixth, because women are more likely to fall and wear different footwear, we specifically recruited older women into the study, but we cannot be certain that the findings are generalisable to men. Finally, as with our previous study [12], our assessment protocol did not include any tests specifically targeting slip resistance, so the slip resistant features of the outersole of the supportive footwear were not evaluated. However, the outersole design features have previously been shown to enhance slip resistance [33–37], and are likely to be superior to those of the minimalist footwear.

## Conclusion

Standing balance and walking stability was similar between supportive and minimalist footwear conditions. Participants did however, perceive the supportive footwear to be more aesthetically pleasing, easier put on and off, comfortable and stable compared to the minimalist footwear. Ongoing research is required to determine whether footwear designed to improve balance and stability, such as ours, can reduce the risk of falls, and prospective studies will need to be conducted to determine longer-term effects of these supportive footwear styles on standing balance and walking stability in older women.

## Data Availability

The datasets used and/or analysed during the current study are available from the corresponding author on reasonable request.
